# Functional performance of patients submitted to cardiac surgery with different levels of sleep quality: an observational study

**DOI:** 10.1016/j.bjorl.2024.101497

**Published:** 2024-09-02

**Authors:** André Luiz Lisboa Cordeiro, Hayssa de Cássia Mascarenhas Barbosa, Daniel Silva Mascarenhas, Jandesson Cena dos Santos, André Raimundo França Guimarães

**Affiliations:** a Escola Bahiana de Medicina e Saúde Pública, Salvador, BA, Brazil; b Centro Universitário Nobre, Feira de Santana, BA, Brazil; c Instituto Nobre de Cardiologia, Feira de Santana, BA, Brazil

**Keywords:** Sleep, Cardiac surgery, Functional performance

## Abstract

**Objective:**

To describe pulmonary function, muscle strength and functional performance in the different qualities of sleep and the impact of this on the number of physiotherapeutic assistances.

**Methods:**

This is an observational study. In the pre and post operative period, sleep behavior was evaluated using the Pittsburgh questionnaire. Patients were divided into three groups: Good Sleep Quality (GSQ), Poor Sleep Quality (PSQ) and Sleep Disordered (SDB). At this time, other tests were also performed, such as: 6-Minute Walk Test (6MWT), Sit and Stand Test (SST), gait speed test and Timed Up to Go (TUG), Medical Research Council (MRC), maximal inspiratory pressure and maximal expiratory, vital capacity and peak expiratory flow. The functional performance and lung function of each group were compared.

**Results:**

A total of 105 people, undergoing cardiac surgery and admitted to hospital were evaluated, 33 with GSQ, 41 with PSQ and 31 with SD. Patients who were in the SDB group had a lower functional performance than the other groups. 6MWT (meters) in the GSQ was 499 ± 87, versus 487 ± 91 in the PSQ and 430 ± 78 in the SD (*p* = 0.02). In the SST (seconds) it was 10.4 ± 1.1 in the GQS, 11.1 ± 2.3 in the PSQ and 15.4 ± 2.1 in the SD (*p* = 0.04). Lung function and muscle strength did not differ between groups. Regarding the refusal to perform physical therapy, the SD group was more incident, the main reason being drowsiness.

**Conclusion:**

Based on the results, we found that sleep quality interferes with functional performance and physical therapy assistance during the hospital stay in patients undergoing cardiac surgery.

## Highlights

• SD in the preoperative period have a lower functional performance.
• Good sleep quality had higher functional performance in functional tests.
• Physical therapy care for patients with sleep disorders difficult.

## Introduction

Post-cardiac surgery patients are commonly referred to the Intensive Care Unit for constant monitoring and assistance. However, the period of hospitalization may favor the worsening of sleep quality, since it refers to a unit that generates stress.^1^, ^2^ This decline can impact on the ability to perform exercises, generating a decrease in functional capacity.

Impasses related to neuromuscular disorders, surgical wounds, changes in appetite, emotional changes, drug therapy and emotional changes can contribute to variations in sleep quality in the Postoperative Period (POD).^3^ Equipment, professionals and procedures contribute to sleep fragmentation and interrupt the circadian cycle, causing damage, for example, increased blood pressure.^4^

Studies have shown that poor sleep quality has affected POD patients, insomnia has been the main related disorder.^5^ Thus, individuals with insomnia tend to have anxiety, daytime sleepiness, muscle pain, irritability, low intellectual performance, fatigue, tiredness and tension.^6^, ^7^, ^8^

Changes associated with cardiac surgery and sleep quality worsen the ability to exercise. Functional performance should be evaluated preoperatively, serving as a postoperative comparison and as a criterion for the progression of mobilization. There are several studies that assess respiratory and peripheral muscle strength and functional capacity after cardiac surgery, however, no studies were found on the different levels of sleep.^9^, ^10^

Thus, the primary objective of this study is to describe pulmonary function, respiratory and peripheral muscle strength and functional performance in the different qualities of sleep. The secondary is to observe if the sleep alteration generates refusal for physical therapy care.

## Methods

### Design of study

This is an observational study, carried out with patients undergoing coronary artery bypass grafting at the Instituto Nobre de Cardiologia in Feira de Santana - Bahia, from January 2018 to February 2020.

### Inclusion and exclusion criteria

The following inclusion criteria were used: Individuals of both sexes, with Coronary Artery Disease (CAD), aged over 18 years and undergoing coronary artery bypass grafting with cardiopulmonary bypass and median sternotomy. The exclusion of intra-aortic balloon use, surgical reintervention, valvular heart disease, previous pulmonary disease, who did not understand how to perform the proposed techniques, presented hemodynamic instability during the evaluation, physical limitation, such as amputation, that compromised the performance of the exercises, patients with difficulties in understanding the proposed questionnaire, bringing incompatibility of information or inability to answer the same question, or who refused to answer it.

### Ethical aspects

Our study was submitted to and approved by the Ethics and Research Committee of Faculdade Nobre de Feira de Santana, obtaining opinion number 4,008,896. All participants signed an informed consent form.

### Study protocol

In the preoperative period, clinical and surgical characteristics such as diabetes mellitus, systemic arterial hypertension, dyslipidemia, acute myocardial infarction and sedentary lifestyle were collected. All these comorbidities were known through the medical records of each patient, with the exception of sedentary lifestyle, where the International Physical Activity Questionnaire (IPAQ) was applied in long format, which evaluates 27 questions related to physical activities performed in a normal week, with light intensity, moderate and vigorous with a continuous duration of 10 min, divided into four categories of physical activity such as work, transportation, domestic activities and leisure. Those who did not perform any physical activity for at least 10 continuous minutes during the week were considered sedentary.^11^

For information on sleep behavior, the Pittsburgh questionnaire was applied, originally developed by Buysse et al.,^12^ which measures retrospective sleep quality and disturbances over a 1-month period for use in clinical practice and research. It discriminates between good and bad sleepers and provides a brief, clinically useful assessment of various sleep disorders.

It consists of 19 questions that are grouped into 7 components, each on a scale graduated from zero (no difficulty) to three (severe difficulty). The PSQI components are: C1 Subjective sleep quality, C2 Sleep latency, C3 Sleep duration, C4 Usual sleep efficiency, C5 Sleep alterations, C6 Medication use, C7 Daytime sleep dysfunction. The sum of the values assigned to the seven components ranges from 0 to 21 in the total score of the questionnaire, indicating that the higher the number, the worse the quality of sleep. Scores of 0–4 indicate good sleep quality, 5–10 indicate poor quality and above 10 indicate sleep disturbances.^12^

In addition to the questionnaire applied, other tests were also performed in the preoperative period, such as: 6-Minute Walk Test (6MWT), Sit and Stand Test (SST), gait speed test, Timed Up to Go (TUG), Maximum Inspiratory Pressure (MIP), Maximum Expiratory Pressure (MEP), Medical Research Council (MRC), Vital Capacity (VC) e Peak Expiratory Flow (PEF).

The participants of the present study were divided into good sleep quality, poor sleep quality and sleep disturbance according to their scores on the Pittburgh questionnaire and consequently, evaluated on each specific test mentioned above. The same questionnaire was administered on the hospital discharge to check the impact of the procedure and hospital stay on sleep quality.

### Evaluated variables

The 6-Minute Walk Test (6MWT) is one of the main tests to assess the patient's submaximal functional capacity, to start the test, the evaluator will need a stopwatch, two cones to demarcate the space, a sphygmomanometer, an automatic external defibrillator and a fast-moving chair. The evaluator instructed the patient on how to perform the test and what type of footwear and clothing to wear for the application. The test was carried out in a 30-meter, flat and obstacle-free corridor. Intense activities should be avoided 2 h before the test. Before starting the test, one should measure Blood Pressure (BP), assess the level of oxygenation using a pulse oximeter, level of dyspnea, Heart Rate (HR) and Respiratory Rate (RR). The patient is instructed to walk as quickly as possible, without running, going around this circuit for 6-min. In specific cases, an oxygen cylinder can be used to increase the patient's tolerance or aids for walking. During the exercise, the patient's encouragement is allowed, always interspersing each minute of the test.^13^

The Sit and Stand Test (SST) was performed through 5 repetitions, reproducing the act of sitting and standing independently. During the test, the use of devices or arms for support was not allowed. The arms should remain crossed over the patient's chest to prevent use. It should be performed as quickly as the patient can, having his time clocked, without any encouragement from the evaluator.^14^

In the Gait Speed Test (TVM), the patient was instructed by the evaluator to walk at their usual speed on a 10-meter track. The evaluator took into account that the initial two meters were for acceleration and the final two meters for deceleration. The time was only timed between the second and the sixth meter. Speed was calculated as follows by dividing the distance in meters by the running time in seconds.^15^

In Time Up and Go (TUG) the patient started the test sitting in a chair without arms and walked a distance of three meters, returned and sat down again in the chair. The time was only recorded from the moment the patient got up from the chair and was paused when he sat down again.^16^

Assessment of inspiratory muscle strength, MIP, was performed using an Indumed® (São Paulo, Brazil) analogue manovacuometer. During the evaluation, a maximal expiration until the residual volume was requested and then a maximal and slow inspiration to the total lung capacity was required; this test was done using the unidirectional valve method, being possible a flow through a hole of one millimeter, aiming to exclude the action of the buccinator and repeated for three times, being used the highest value reached, as long as this value was not the last. MEP was evaluated using the same apparatus and the patient was instructed to perform a maximal inspiration until he reached his total pulmonary capacity, the mask was placed and after that a maximum expiration was requested until the residual capacity was reached. The test was repeated three times and it was considered the highest value result, as long as this value was not the last.^17^

To assess VC, it was used the analogue ventilometer Ferraris Mark 8 Wright Respirometer (Louisville, Colorado, Unite States of America). The ventilometer was unlocked, cleared and soon after the facial mask was placed on the face of the individual. The patient underwent deep inspiration until he/she reached his/her total pulmonary capacity and soon after a slow and gradual expiration until reaching the residual volume. After this, the ventilometer was locked and the result observed and noted. The test was repeated three times, being considered the highest value result.^18^

Peak expiratory flow was evaluated using the peak flow of the Mini Wright® brand. During the evaluation, the patient was seated, with his head in a neutral position and a nasal clip to prevent air from escaping through the nostrils. The patient took a deep breath, until total pulmonary capacity, followed by forced expiration with the mouth in the device. After three measurements, the highest value was chosen and there could be no difference > 40 L between measurements.^19^

Each test was performed twice, the first attempt was considered learning, the second evaluative attempt and they were performed by a trained evaluator blinded to the research.

### Statistical analysis

SPSS version 20.0 software was used for data analysis. Normality was verified using the Shapiro-Wilks test. Continuous variables were expressed as mean and standard deviation. The variables were checked between the three groups using ANOVA. It was considered significant if the *p*-value < 0.05.

## Results

**Figure 1:**
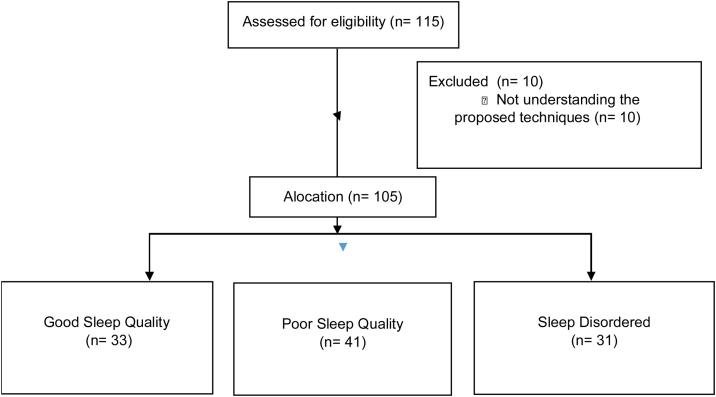
Flowchart of the division of groups based on the Pittsburgh questionnaire (PSQI) in patients undergoing coronary artery bypass grafting.

Participated in the study 105 people undergoing cardiac surgery divided into three groups. Good Sleep Quality (GSQ) 33 patients, Poor Sleep Quality 41 patients and Sleep Disorder (SD) 31 patients (Fig. 1). Of the total number of patients, 57 (54%) were men, aged 65 ± 5 years. In addition, more than half had a history of sedentary lifestyle 65 (62%) and pre-existing factors such as arterial hypertension 58 (55%), diabetes mellitus 46 (44%). The other data are shown in Table 1.

**Table 1:** Clinical and surgical data of the patients studied.

Variable	GSQ (*n* = 33)	PSQ (*n* = 41)	SD (*n* = 31)	*p*^a^
Age (years)	62 ± 6	65 ± 5	67 ± 5	0.43
Gender				
Male	18 (55%)	22 (54%)	17 (55%)	
Female	15 (45%)	19 (46%)	14 (45%)	0.65
BMI (kg/m^2^)	24 ± 3	26 ± 3	26 ± 4	0.54
History of Smoking	4 (12%)	4 (10%)	5 (16%)	0.48
Ejection Fraction	52 ± 4	53 ± 5	53 ± 4	0.73
Physical Activity Level				0.81
Active	13 (39%)	15 (37%)	12 (39%)	
Sedentary	20 (61%)	26 (63%)	19 (61%)	
Comorbidities				
SAH	19 (58%)	21 (51%)	18 (58%)	0.59
DM	14 (42%)	17 (41%)	15 (48%)	0.43
DLP	13 (39%)	15 (37%)	13 (39%)	0.81
Surgery time (hours)	4.2 ± 1.3	4.3 ± 1.5	4.3 ± 1.6	0.79
CPB time (min)	87 ± 10	90 ± 12	89 ± 12	0.79
Length of stay in the ICU (days)	2 ± 1	2 ± 2	3 ± 2	0.32
Length of hospital stay (days)	10 ± 2	9 ± 4	11 ± 3	0.58
MV time (hours)	7 ± 2	8 ± 2	7 ± 3	0.69
Number of drains	2 ± 1	2 ± 1	2 ± 1	0.95
Number of grafts	2 ± 1	2 ± 1	2 ± 1	0.94

BMI, Body Mass Index; CPB, Cardiopulmonary Bypass; SAH, Systemic Arterial Hypertension; DM, Diabetes Mellitus; DLP, Dyslipidemia; ICU, Intensive Care Unit; MV, Mechanical Ventilation; GSQ, Good Sleep Quality; PSQ, Poor Sleep Quality; SD, Sleep Disorder.

a ANOVA.

**Figure 2:**
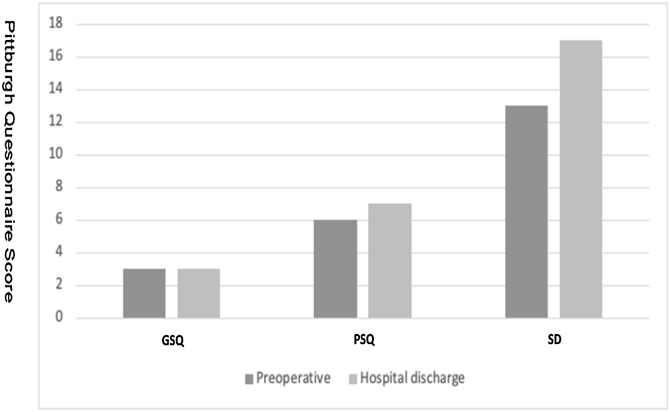
Behavior of sleep quality in the preoperative period and hospital discharge. GSQ, Good Sleep Quality; PSQ, Poor Sleep Quality; SD, Sleep Disorder.

Fig. 2 shows patients in the pre and postoperative period of cardiac surgery, the PQS group showed no difference, the GQS group had a slight difference, and the SD group had a 17% increase in the score.

Table 2 shows the functional performance of the patients studied and those who had SD in the preoperative period have a lower functional performance. Patients with GSQ walked more in the 6MWT with 499 m, PSQ walked 487 m and SD 430 m (*p* =  0.02). The QSB group presented a shorter time in the SST with 10.4 s, QSR presented 11.1 s and DS presented 15.4 s (*p* =  0.04).

**Table 2:** Performance in functional tests according to the studied group.

Variável	GSQ (*n* = 33)	PSQ (*n* = 41)	SD (*n* = 31)	*p*^a^
6MWT (meters)	499 ± 87	487 ± 91	430 ± 78	0.02
SST (seconds)	10.4 ± 1.1	11.1 ± 2.3	15.4 ± 2.1	0.04
Gait speed (m/s)	1.4 ± 0.6	1.2 ± 0.8	1.0 ± 0.8	0.23
TUG (seconds)	8.9 ± 1.1	9.2 ± 1.5	11.5 ± 1.2	0.04
MRC	58 ± 1	59 ± 1	57 ± 2	0.76
MIP (cm H_2_O)	112 ± 5	115 ± 3	113 ± 4	0.69
MEP (cm H_2_O)	102 ± 6	105 ± 5	108 ± 7	0.52
VC (mL/kg)	55 ± 2	53 ± 2	55 ± 3	0.43
PEF (L/min)	412 ± 37	405 ± 30	401 ± 44	0.32

6MWT, Six-Minute Walk Test; SST, Sit and Stand Test; VM, Gait speed; TUG, Timed Up and Go; GSQ, Good Sleep Quality; PSQ, Poor Sleep Quality; SD, Sleep Disorder.

a ANOVA.

Table 3 shows that the patients studied with SD had a significant amount of refusal in physical therapy due to drowsiness (*p* <  0.01). The total number of physical therapy visits were 738 in the GSQ group, 1111 PSQ and 1034 SD.

**Table 3:** Reasons associated with the patient's refusal to perform the Physiotherapy session.

Variable	GSQ (*n* = 33)	PSQ (*n* = 41)	SD (*n* = 31)	*p*^a^
Total physical therapy assistance that could be performed	738	1111	1034	<0.01
Total assistance performed	98%	98%	98%	0.91
Fear	2 (0.2%)	2 (0.2%)	2 (0.2%)	0.89
Pain	3 (0.4%)	2 (0.2%)	3 (0.3%)	0.72
Somnolence	1 (0.1%)	3 (0.3%)	9 (0.9%)	<0.01
Insecurity	2 (0.2%)	3 (0.3%)	3 (0.3%)	0.82
Malaise	3 (0.4%)	2 (0.2%)	3 (0.3%)	0.69
Others	3 (0.4%)	3 (0.3%)	3 (0.3%)	0.94

GSQ, Good Sleep Quality; PSQ, Poor Sleep Quality; SD, Sleep Disorder.

a ANOVA.

## Discussion

Our study showed that individuals with good sleep quality had higher functional performance in the six-minute walk test and in the sit-to-stand test. In addition, drowsiness was one of the reasons for recourse that made physical therapy care for patients with sleep disorders difficult.

The Sleep Disorder group showed low performance when compared with the other groups that performed the same tests. In the sit and stand test, TUG and 6MWT, the performance of this group was inferior to the others. Thus, a functional decline in lower limb strength, decreased balance, risk of falls, hospital readmission and functional performance.^20^, ^21^ Thus, we list that patients undergoing cardiac surgery in the preoperative period who present SD may have a functional decline and worsening of sleep quality according to their hospital stay. This finding corroborates the study by Liu et al.^3^ who reported a percentage of poor sleep quality already in the preoperative period, leading to an increase in the DS rate at hospital discharge of these individuals.

Patients with SD tend to have excessive daytime sleepiness, in our study, the population analyzed was elderly and such individuals have difficulties with sleep due to aging. Kater-el et al.^22^ mention the sleep disorder as a trigger for the triggering of fatigue, resulting from an age-dependent pro-inflammatory state, in addition to other variables such as sleep disorder, arteriosclerosis, obesity and cancer, decreasing the level of functionality and mobility in the elderly. However, there is a scarcity of studies that evaluate the functional performance of patients in the preoperative period of cardiac surgery relating it to sleep quality, thus, the following scientific evidence contributes to the findings of this study regarding sleep quality and the surgical cardiovascular procedure.

According to Beck et al.^23^ individuals undergoing cardiac surgery have excessive daytime sleepiness, evidencing the existence of changes in sleep quality, which contributes to the results of the present study, such changes in quality of sleep occur by cycles of sleep interrupted by factors present in the hospital environment already mentioned above. According to Wesselius et al.,^24^ the hospital environment can be harmful both in the duration and in the quality of sleep of patients who receive assistance in this place. Cardiac surgery patients experience discomfort, pain, decreased sleep depth, which together with other factors such as alarms, voices and interventions influence poor sleep quality within this unit.^2^

According to Kamar et al.^25^ sleep is a health need and, given that it has an adequate duration, quality, regularity and absence of disturbances, it contributes to physical, mental and emotional health. Sleeping less than five to six hours contributes to increased cortisone secretion, decreased glucose intolerance and increased sympathetic activity that are associated with reduced hours of sleep.^26^ With activation of the sympathetic, autonomic system, noradrenaline levels increase in the body and are directed through the sympathetic and post ganglionic nerves, increasing release rates at nerve terminals and directing their flow to internal organs, including the heart and kidneys, which are evident mechanisms in Systemic Arterial Hypertension (SAH). Being, noradrenaline the main neurotransmitter involved in this correlation between sympathetic activity and SAH.^27^ In our study, patients in the PSQ group had a higher prevalence of SAH, a factor that influences physiological changes in this individual profile.

Javanmard et al.^28^ in their research, mentions about the patient undergoing CABG and oxidative stress due to the surgical procedure, in addition, showing that individuals with coronary heart disease have a low production of melatonin. As melatonin is secreted by the pineal gland, it plays a significant role in the circadian cycle, in addition to reducing free radicals. It also stabilizes the inner membranes of mitochondria and decreases electron and free radical leakage.

Although patients in the PSQ group had a higher SAH index, the other variables were very close to the groups with sleep disorders and without poor sleep quality. Such variables may not show significance, because the groups are heterogeneous with both sexes, in addition to not being evaluated the levels of fatigue and collection of laboratory tests. However, the literature presents a view focused on ventilatory and peripheral muscle strength, in addition to ventilatory parameters, since such factors are essential in post-cardiac surgery rehabilitation and are associated with complications.^29^ The lack of studies that assess different levels of sleep quality highlights the importance of our study, corroborating the relationship between heart surgery and different levels of sleep.

Some studies have linked factors related to the worsening of sleep quality in the ICU, however, what has hampered the implementation of therapy and improvement of these patients is the quality of sleep associated with emotional factors such as anxiety.^30^, ^31^ In our study, patients with Sleep Disorder showed greater refusal to physical therapy care. Another correlated variable was sleepiness having a higher index and significance in patients with SD. Individuals with excessive daytime sleepiness have changes in the circadian cycle, sleep disturbances, and chronic diseases such as hypertension and diabetes.

These findings corroborate previous studies that have also found an association between sleep disturbances and lower functional performance after cardiac surgery.^32^, ^33^ In addition, the higher incidence of refusal of physical therapy in the SD group, attributed mainly to sleepiness, echoes previous findings that highlighted the relationship between excessive daytime sleepiness and lower adherence to post-surgical rehabilitation.^34^, ^35^

However, it is important to note that other studies have found contradictory results regarding the association between sleep disturbances and functional performance after cardiac surgery. For example, a recent meta-analysis study found no significant differences in functional performance between patients with and without sleep disorders after cardiac surgery.^36^ In addition, some studies suggest that factors such as age, comorbidities and postoperative recovery time may play a crucial role in determining functional performance, regardless of the presence of sleep disorders.^37^, ^38^

However, having a clinical perspective beyond conventional physiotherapy contributes to specialized and individual assistance regarding the treatment of these patients. In order to reduce the stressors inside the intensive care unit and the length of stay. Based on the results, we found that sleep quality interferes with functional performance and daytime sleepiness in patients undergoing cardiac surgery. In addition to impairing the number of physiotherapeutic visits, the group with SD had a worse outcome in the preoperative period until hospital discharge.

The limitations of the present study include the lack of evaluation of the participants in the postoperative period of cardiac surgery regarding functional performance. Another limitation was the absence of information about sleep disorder diagnosis and medication use. The absence of a sample calculation can affect the outcome of this work in several ways. Firstly, the lack of a determined sample size can result in a sample that is not statistically representative of the target population, which can lead to biased or inaccurate conclusions. Furthermore, the lack of statistical power due to an inadequate sample can impact on the ability to detect statistically significant differences between the groups studied. In the specific case of the results presented, the significant difference in functional performance between the groups could be questioned if there was no sample calculation carried out beforehand.

When assessing sleep quality in patients undergoing cardiac surgery using questionnaires, it is important to recognize and highlight the bias potentially present in this method of categorization. The results of this study revealed significant differences in functional performance between the groups of patients classified with different sleep qualities. However, it is crucial to recognize that questionnaires used to assess sleep can be susceptible to self-reporting biases, influenced by various factors, such as the patient's subjective perception of sleep and their ability to accurately recall sleep patterns. In addition, other aspects not addressed by the questionnaires, such as undiagnosed sleep disorders or underlying medical conditions, can influence the results. Therefore, although the findings of this study provide valuable insights into the relationship between sleep quality and post-surgical functional performance, it is essential to interpret them with caution, recognizing the inherent limitations of the assessment methods used.

## Conclusion

Based on the results, we found that sleep quality interferes with functional performance and daytime sleepiness in patients undergoing cardiac surgery, in addition to having an impact on the amount of physical therapy visits during the hospital stay.

These findings pointing to the interference of sleep quality on functional performance, daytime sleepiness and frequency of physiotherapy visits in patients undergoing cardiac surgery, a multifaceted line of research is suggested to explore and address this phenomenon. This could involve developing specific interventions to improve the sleep of these patients, investigating the mechanisms underlying the relationship between sleep and post-surgical recovery, identifying high-risk patients for sleep disorders and developing multidisciplinary approaches that integrate mental health professionals, physiotherapists and sleep specialists. In addition, the use of advanced sleep monitoring technologies and the assessment of the long-term effects of sleep quality in this population can provide valuable insights for improving clinical outcomes and quality of life for patients after cardiac surgery.

The authors state that the data is available for consultation.

## Funding

No financing has been acquired.

## Conflicts of interest

The authors declare no conflicts of interest.
